# Draft Genome Sequence of *Sphingobium* sp. Strain HDIPO4, an Avid Degrader of Hexachlorocyclohexane

**DOI:** 10.1128/genomeA.00749-13

**Published:** 2013-09-19

**Authors:** Udita Mukherjee, Roshan Kumar, Nitish Kumar Mahato, J. P. Khurana, Rup Lal

**Affiliations:** Department of Zoology, University of Delhi, Delhi, Indiaa; Interdisciplinary Centre for Plant Genomics & Department of Plant Molecular Biology, University of Delhi South Campus, New Delhi, Indiab

## Abstract

*Sphingobium* sp. strain HDIPO4 was isolated from a hexachlorocyclohexane (HCH) dumpsite and degraded HCH isomers rapidly. The draft genome sequence of HDIPO4 (~4.7 Mbp) contains 143 contigs and 4,646 coding sequences with a G+C content of 65%.

## GENOME ANNOUNCEMENT

*Sphingobium* sp. strain HDIPO4 was originally isolated in 2008 (1) from the hexachlorocyclohexane (HCH)-contaminated dumpsite at Ummari Village, Lucknow, India. Phylogenetic analysis using 16S rRNA gene sequences showed that strain HDIPO4 is most closely related to another HCH-degrading strain, *Sphingobium francense* Sp+ (with 98% sequence similarity). Strain Sp+ was previously isolated from French soil contaminated with γ-HCH (2, 3). In contrast to strain Sp+, which degrades HCH isomers slowly, strain HDIPO4 was found to degrade HCH isomers at a high rate (1).

The genome sequencing of strain HDIPO4 was carried out by using the Illumina genome analyzer platform and the 454 GS FLX Titanium platform. For this purpose, paired-end libraries of 2 kb were constructed that led to the generation of 761 Mbp of raw data, which generated 561 Mbp of clean data.

The draft genome sequence of HDIPO4 consists of 4,741,576 bp with 90× genome coverage. The draft genome was assembled into 143 contigs by using the ABySS 1.3.3 assembler (4) at a k-mer of 53 with an average G+C content of 65%. The validation of the final assembly was done based on the paired-end information. The annotations were done by using RAST version 4.0 (5) and the NCBI Prokaryotic Genomes Annotation Pipeline (PGAP) version 2.0 (http://www.ncbi.nlm.nih.gov/genomes/static/Pipeline.html).

A total of 4,646 open reading frames (ORFs) and 54 tRNA and 13 rRNA genes were identified. In addition, 3 copies of the 16S rRNA gene were also detected. Interestingly, this strain contained two copies each of *linB* (upper degradation pathway genes) and *linDER* (lower degradation pathway genes) (6), in contrast to previously reported sphingomonads that contained only one copy each of these genes (7, 8). The presence of two copies each of *linB* and *linDER* may be responsible for the faster degradation of HCH isomers by this strain.

Besides the HCH degradation pathway genes, strain HDIPO4 showed the presence of phenol-, chlorophenol-, toluene-, and homogentisate-degrading gene clusters. Eighteen copies of IS*6100* were found that are known to play active roles in horizontal gene transfer of *lin* genes among sphingomonads (3). The TonB-dependent receptor, which mediates transport of iron siderophore complexes in Gram-negative bacteria (11), was also detected in abundance, in the vicinity of the *lin* genes. A total of 29 insertion sequence (IS) elements, including IS*6100*, ISSp*5*, ISGbe*1*, and ISSpma*1*, were also present in the draft genome (12).

Such myriad *lin* genes, transposons, and other catabolic genes were also reported in the genomes of HCH-degrading sphingomonads (7–10) and as products of enrichment and colossal horizontal gene transfer in the recent metagenomic analyses of the HCH dumpsite (13, 14). The genome sequence of this strain, especially the presence of multiple copies of *lin* genes, clearly indicates that it may be useful in the development of a consortium for bioremediation of HCH at the HCH dumpsite.

### Nucleotide sequence accession numbers.

The genome sequence of *Sphingobium* sp. HDIPO4 has been assigned GenBank accession number ATDO00000000. The version described in this paper is the first version, ATDO01000000.

## References

[1] Dhadwal M Singh A Prakash O Gupta SK Kumari K Sharma P Verma M Holliger C Lal R 2008 Proposal of biostimulation for hexachlorocyclohexane (HCH)-decontamination and characterization of culturable bacterial community from high-dose point HCH-contaminated soils. J. Appl. Microbiol. 106:381–392 10.1111/j.1365-2672.2008.03982.x 19200306

[2] Pal R Bala S Dadhwal M Kumar M Dhingra G Prakash O Prabagaran SR Shivaji S Cullum J Holliger C Lal R 2005 Hexachlorocyclohexane-degrading bacterial strains *Sphingomonas paucimobilis* B90A, UT26 and Sp+, having similar *lin* genes, represent three distinct species, *Sphingomonas indicum* sp. nov., *Sphingobium japonicum* sp. nov., and *Sphingobium francense* sp. nov., and reclassification of *Sphingomonas chungbukensis* as *Sphingobium chungbukense* comb. nov. Int. J. Syst. Evol. Microbiol. 55:1965–1972 16166696 10.1099/ijs.0.63201-0

[3] Dogra C Raina V Pal R Suar M Lal S Gartemann KH Holliger C van der Meer JR Lal R 2004 Organization of *lin* genes and IS*6100* among different strains of hexachlorocyclohexane-degrading *Sphingomonas paucimobilis*: evidence for horizontal gene transfer. J. Bacteriol. 186:2225–2235 15060023 10.1128/JB.186.8.2225-2235.2004 PMC412113

[4] Simpson JT Wong K Jackman SD Schein JE Jones SJ Birol I 2009 ABySS: a parallel assembler for short read sequence data. Genome Res. 19:1117–1123 19251739 10.1101/gr.089532.108 PMC2694472

[5] Aziz RK Bartels D Best AA DeJongh M Disz T Edwards RA Formsma K Gerdes S Glass EM Kubal M Meyer F Olsen GJ Olson R Osterman AL Overbeek RA McNeil LK Paarmann D Paczian T Parrello B Pusch GD Reich C Stevens R Vassieva O Vonstein V Wilke A Zagnitko O 2008 The RAST server: rapid annotations using subsystems technology. BMC Genomics 9:75 18261238 10.1186/1471-2164-9-75 PMC2265698

[6] Lal R Pandey G Sharma P Kumari K Malhotra S Pandey R Raina V Kohler HP Holliger C Jackson C Oakeshott JG 2010 Biochemistry of microbial degradation of hexachlorocyclohexane and prospects for bioremediation. Microbiol. Mol. Biol. Rev. 74:58–80 20197499 10.1128/MMBR.00029-09 PMC2832351

[7] Anand S Sangwan N Lata P Kaur J Dua A Singh AK Verma M Kaur J Khurana JP Khurana P Mathur S Lal R 2012 Genome sequence of *Sphingobium indicum* B90A, a hexachlorocyclohexane-degrading bacterium. J. Bacteriol. 194:4471–4472 22843598 10.1128/JB.00901-12 PMC3416241

[8] Niharika N Sangwan N Ahmad S Singh P Khurana JP Lal R 2013 Draft genome sequence of *Sphingobium chinhatense* strain IP26^T^, isolated from a hexachlorocyclohexane dumpsite. Genome Announc. 1(4):e00680-13. 10.1128/genomeA.00680-13 23990581 PMC3757456

[9] Saxena A Nayyar N Sangwan N Kumari R Khurana JP Lal R 2013 Genome sequence of *Novosphingobium lindaniclasticum* LE124^T^, isolated from a hexachlorocyclohexane dumpsite. Genome Announc. 1(5):e00715-13. 10.1128/genomeA.00715-13 24029761 PMC3772145

[10] Singh AK Sangwan N Sharma A Gupta V Khurana JP Lal R 2013 Draft genome sequence of *Sphingobium quisquiliarum* P25^T^, a novel hexachlorocylohexane (HCH)-degrading bacterium isolated from an HCH dumpsite. Genome Announc. 1(5):e00717-13. 10.1128/genomeA.00717-13 24029763 PMC3772147

[11] Miller TR Delcher AL Salzberg SL Saunders E Detter JC Halden RU 2010 Genome sequence of the dioxin-mineralizing bacterium *Sphingomonas wittichii* RW1. J. Bacteriol. 192:6101–6102 20833805 10.1128/JB.01030-10 PMC2976435

[12] Siguier P Perochon J Lestrade L Mahillon J Chandler M 2006 ISfinder: the reference centre for bacterial insertion sequences. Nucleic Acids Res. 34:D32–D36. 10.1093/nar/gkj014 16381877 PMC1347377

[13] Sangwan N Lata P Dwivedi V Singh A Niharika N Kaur J Anand S Malhotra J Jindal S Nigam A Lal D Dua A Saxena A Garg N Verma M Kaur J Mukherjee U Gilbert JA Dowd SE Raman R Khurana P Khurana JP Lal R 2012 Comparative metagenomic analysis of soil microbial communities across three hexachlorocyclohexane contamination levels. PLoS One 7:e46219. 10.1371/journal.pone.0046219 23029440 PMC3460827

[14] Sangwan N Verma H Kumar R Negi V Lax S Khurana P Khurana JP Gilbert JA Lal R Reconstructing an ancestral genotype of two hexachlorocyclohexane degrading *Sphingobium* species using metagenomic sequence data. ISME J., in press 10.1038/ismej.2013.153 PMC3906814 24030592

